# Professional Influence upon Legislation

**Published:** 1915-03

**Authors:** 


### Professional Influence Upon Legislation.

One frequently hears pessimistic expressions as to the lack of influence of the medical profession in regard to legislation. To a certain degree, these are warranted but, considering the successful efforts of a much divided profession to secure educational requirements both for matriculation and license, to combat the anti-vivisectionists, to secure the enlargement and efficiency of health departments and the like, the profession may feel reasonably well satisfied.

One fact must not be overlooked: all legislative bodies must be as impartial as possible, they must refuse to pass anything like class legislation unless clearly shown to be justified by the welfare of the community. Even if they could look at matters from the standpoint of a specially, technically, educated profession, they are obliged to be guided largely by popular sentiment. If, for any reason, the profession can not secure this support, the fault does not lie with the legislative body but either with the profession or the people. On the other hand, some cynic may remind us that legislatures sometimes respond to arguments that can not be publicly used. The profession never has and never can, employ bribes or appeal indirectly to the self-interest of legislators, except as their own interests are the same as any citizen's.

What particularly concerns the medical profession, looking at the matter broadly and with regard to obtaining the best results in the future, is itself, not the legislatures nor the people. What are the strong points, what the weak points in ourselves?

The strong points, so far as we can estimate them in general terms are four: 1. Obviously, the medical profession is better able to judge on matters in which it is interested to obtain legislation than anyone else; 2. If is fairly well organized, much better than ever before in this country, unless perhaps early in the last century; 3. More than almost any other body of men or women who appeal to legislatures, it is intelligent, of good standing in the community, unselfish and hard headed; 4. It has an established precedent of asking legislation in the interests of the people themselves.

The weak points may, with possible individual exceptions, be summarized as merely failure of perfection in the strong points. For example, while the medical profession naturally has the best obtainable knowledge on sanitary, hygienic and many kinds of sociologic problems requiring legislation, it must be admitted that this knowledge is not absolutely infallible and the very frankness with which the profession acknowledges its errors, changes its view point according to actual demonstration and endeavors to avoid obstinate devotion to disproved theories, has given an impression of vacillation and ignorance. We can recall the importance attached to ice, flies, etc., as causative factors of typhoid; the sporadic enthusiasm as to hypnotism, psycho-therapy and the like; the sudden change of front with regard to the contagiousness of yellow fever, the paradox of a demand for special institutions for the tuberculous, by men much divided among themselves as to the actual direct communicability of the disease and forced to admit that excellent results have been obtained in very different climates.

Secondly, while the combining of forces by the two factions of the regular profession in New York and the very general co-operation and reunion of the Homoeopaths, as well as the phenomenal growth in numbers and solidarity of the American Medical Association and its states and county affiliations, have very materially strengthened the profession, the same centripetal forces have called into action centrifugal forces almost equally great. The very legislation that we have secured in regard to educational standards, and against quackery, and the tendency to consolidation, has led to the formation of almost equally organized schools of special treatment which, from the legislative standpoint, must be treated with the same degree of consideration as ourselves. Again, professional solidarity has been accomplished at the cost of much internal dissent, distrust and open hostility. For instance, a county society recently passed a measure in regard to which it was recognized that many questions might arise requiring individual decision. A qualification to this effect was voted down, almost unanimously, on the explicit argument that it would be used by influential physicians to further their own selfish ends. Now, when such an argument is raised by physicians themselves, against their own fellows and is accepted by vote, what can we say to the people or the legislature that questions our sincerity?

In spite of such examples, and the cynic remarks often made by us, we do have, generally speaking, the respect and confidence of the majority of the people. But, so also do Christian Scientists, optometrists, osteopaths, humane societies and similar bodies with whom we often come into conflict. Clothes and manners are commonly spoken of as trivial details; practically, they have great weight as every physician addressing his young confreres at graduation, recognizes. In 1907, at Boston, there was a great gathering of Christian Scientists immediately following the meeting of the American Medical Association. Frankly, which group of women present on account of those two meetings, would, by their general appearance, create the more favorable impression?

We call ourselves a learned profession but it is less than 20 years since we have required any greater education than is customary among clerks, stenographers, or business and professional workers generally above the actual laboring class and our present requirements are by no means so high as those of many business and professions. For many years there was an enormous influx into our ranks, beyond any possible economic demand, of very inadequately prepared men. Now, while many of these men have "made good," both financially and technically and have even educated themselves in later life, and while as individuals, they may have won the respect and trust of their clienteles, we can not get away from the fact that the class of men to whom we must appeal for legislation, includes many of the highest scholastic training and many others who hold it in even greater esteem than it deserves, on account of their own lack.

On the whole, it seems that the medical profession really exerts a considerable influence on legislation and this may be magnified by a candid recognition of the weak points in our own defensive and offensive armor.

